# MAPLE Deposition of Resorbable Calcium Phosphates on Electrospun Nylon Nanofibres for Bone Tissue Engineering

**DOI:** 10.3390/ma19112375

**Published:** 2026-06-03

**Authors:** Andreea Trifan, Gianina Popescu-Pelin, Roxana-Cristina Popescu, Doru-Daniel Cristea, Eduard Liciu, Cristina Busuioc

**Affiliations:** 1 Faculty of Chemical Engineering and Biotechnology, National University of Science and Technology Politehnica Bucharest, 011061 Bucharest, Romania; andreea.trifan@umfcd.ro; 2 3D Printing Laboratory, Center of Innovation and e-Health, “Carol Davila” University of Medicine and Pharmacy, 020021 Bucharest, Romania; 3 National Institute for Laser, Plasma and Radiation Physics, 077125 Magurele, Romania; gianina.popescu@inflpr.ro; 4 Faculty of Medical Engineering, National University of Science and Technology Politehnica Bucharest, 011061 Bucharest, Romania; 5 “Horia Hulubei” National Institute of Physics and Nuclear Engineering, 077125 Magurele, Romania; 6 REOROM Laboratory, Hydraulics Department, National University of Science and Technology Politehnica Bucharest, 060042 Bucharest, Romania

**Keywords:** nanofibres, nylon, calcium phosphates, MAPLE deposition, electrospinning, bone tissue engineering, bioactive coatings, scaffolds, composite materials

## Abstract

One-dimensional fibrous scaffolds with tunable bioactivity offer promise for bone tissue regeneration, yet optimal calcium phosphate phases for enhancing osteogenic performance remain underexplored. This study aimed to evaluate the impact of monetite-, brushite-, and cerium-doped phosphate deposition on electrospun nylon nanofibres functionalised via matrix-assisted pulsed laser evaporation (MAPLE). Five nylon fibre compositions were synthesised, coated with three calcium phosphate phases, and calcined at varying temperatures (500–800 °C) before laser deposition. Physicochemical properties were assessed using energy-dispersive X-ray spectroscopy (EDS), scanning electron microscopy (SEM), and fibre diameter measurements, averaging 62.1±23.8 nm. Biocompatibility assays following MC3T3 preosteoblast seeding and incubation evaluated biological performance. EDX confirmed homogeneous phase deposition; SEM showed phase- and temperature-dependent morphology, with monetite yielding uniform granular structures and cerium-doped phosphate at 800 °C forming dense aggregates. Brushite-coated fibres exhibited superior preosteoblast metabolic activity, reaching 178±2% after 48 h (*p* < 0.001), indicating phase-specific stimulation of bone cell growth. These phosphate-functionalised nylon fibres retain structural integrity, hierarchical porosity, and enhanced bioactivity, providing a versatile electrospinning-MAPLE platform for customisable bone grafts with clinical potential.

## 1. Introduction

Bone defects resulting from trauma, disease, or surgical resection represent a significant clinical challenge [1], with more than two million bone graft procedures performed annually worldwide [2]. While autologous and allogeneic bone grafts remain the gold standard, they are constrained by donor site morbidity, limited tissue availability, immunogenic responses, and disease transmission risk [3]. Consequently, engineered biomaterials designed to recapitulate not only the hierarchical structure but also the dynamic bioactivity of native bone are urgently needed to address this unmet clinical demand [4]. The extracellular matrix (ECM) of bone is fundamentally a composite structure combining a collagenous organic phase with an inorganic mineral phase composed predominantly of calcium phosphate apatite [5]. This hierarchical organisation, spanning scales from nanometres to millimetres, provides mechanical resilience, load transfer, and cell-instructive biochemical cues [6]. Scaffolds designed for bone tissue engineering must therefore reproduce this multiscale architecture, maintain interconnected porosity [7] for nutrient diffusion and vascular ingrowth [8], and present osteoconductive surfaces [9] capable of supporting progenitor cell recruitment and differentiation [10].

Electrospun nanofibres have emerged as a powerful platform for recreating the nano- to microstructure of the ECM [11]. They offer precise control over fibre diameter [12] in the submicron regime, very high surface area-to-volume ratio for protein adsorption and cell interaction [6], tunable pore size and orientation through adjustment of process parameters and collector design [13], and compatibility with a wide range of natural and synthetic polymers that can be blended, functionalised, or loaded with bioactive agents [14].

Natural polymers such as collagen [15] and gelatine provide inherent cell-recognition motifs [16] but often display limited mechanical strength and rapid, difficult-to-control degradation [17]. In contrast, synthetic polymers afford superior mechanical tunability and degradation control, yet they are typically bioinert and require additional strategies to introduce osteogenic signalling [14]. Nylon (polyamide) represents a compelling compromise [18]. Its repeating amide bonds structurally resemble peptide linkages found in proteins [19], which supports good biocompatibility and relatively low immunogenicity, while its mechanical robustness and thermal stability enable the reproducible fabrication of defect-free nanofibres across broad electrospinning windows [20]. Nylon has already been used in sutures, catheters [21] and preliminary bone tissue engineering constructs [12], but systematic studies addressing how different bioactive mineral coatings modulate its performance as a bone scaffold are still scarce [12].

Calcium phosphates (CaPs) are the most physiologically relevant inorganic phases for bone regeneration [22], as their composition closely mirrors the mineral content of native bone [23]. Among them, monetite (CaHPO_4_, anhydrous dicalcium phosphate), brushite (CaHPO_4_·2H_2_O, hydrated dicalcium phosphate) and related orthophosphate (Ca_3_(PO_4_)_2_) and pyrophosphate (Ca_2_P_2_O_7_) phases exhibit distinct solubility, resorption and ion-release profiles, which translate into different osteogenic responses [24]. Monetite possesses higher solubility than hydroxyapatite, enabling sustained calcium and phosphate release and relatively fast but controlled resorption [25]. Brushite resorbs even more rapidly, but it can transform in vivo into less soluble apatite, altering its long-term behaviour [26]. Rare-earth-doped CaP, such as cerium-substituted phosphate [11], retains the osteoconductivity of CaP while introducing additional antioxidant [27] and antimicrobial functionalities arising from the redox activity of cerium ions [28]. These compositional levers make CaP phases attractive candidates for tailoring the biological performance of otherwise bioinert nanofibrous scaffolds [11].

To exploit these phases effectively, deposition techniques must integrate minerals onto delicate polymer fibres without damaging their morphology [29]. Matrix-assisted pulsed laser evaporation (MAPLE) [30] has recently gained attention as a gentle, highly adaptable method for this purpose [31]. Unlike conventional pulsed laser deposition, which directly ablates a solid target and can induce significant thermal and mechanical stress on heat-sensitive substrates, MAPLE employs a frozen target in which the solute of interest is dispersed in a volatile solvent [32]. Upon laser irradiation, the solvent predominantly absorbs the energy [33], undergoes rapid sublimation and entrains the solute species toward the substrate in a plume, where they condense as a thin coating [34]. This indirect transfer mechanism minimises substrate heating, preserves polymer integrity, and allows deposition on three-dimensional architectures such as electrospun mats, including their internal fibre junctions and shadowed regions [28]. Furthermore, MAPLE readily accommodates multicomponent targets, enabling deposition of doped CaP phases or composite organic–inorganic layers with controlled thickness and composition [35].

A variety of strategies have been developed to deposit CaP coatings [36] onto polymer substrates, including biomimetic mineralisation in simulated body fluid [37], sol–gel processing, dip-coating [29] followed by thermal treatment [2], and plasma spraying for high-temperature-resistant polymers. However, these conventional approaches each have important drawbacks: biomineralisation methods are often slow and yield coatings with limited control over thickness and homogeneity, sol–gel and dip-coating routes require post-deposition heat treatments that are incompatible with many biopolymers, and plasma spraying operates at high temperatures and struggles to uniformly coat irregular, three-dimensional architectures. In contrast, MAPLE offers gentle, room-temperature and controlled deposition [28], enabling conformal coatings on complex structures [27].

In this context, the present study systematically evaluates how the controlled deposition of monetite-, brushite- and cerium-doped CaP phases onto electrospun nylon nanofibres via MAPLE affects scaffold architecture, compositional uniformity and early osteogenic performance. By combining structurally robust, ECM-mimetic nylon nanofibres with phase-specific CaP coatings produced by a gentle laser-based method, this work aims to establish a tunable platform for designing next-generation fibrous bone grafts tailored to different clinical requirements.

## 2. Materials and Methods

### 2.1. Materials

Nylon 66 (*Mw* ≈ 15,000–30,000 Da), dimethyl sulfoxide (DMSO, (CH_3_)_2_SO), hexamethyldisilazane (HMDS) and ethanol were purchased from Sigma-Aldrich Co. (Steinheim, Germany). Formic acid (99.5%) and the inorganic precursors calcium nitrate tetrahydrate (Ca(NO_3_)_2_·4H_2_O), diammonium hydrogen phosphate ((NH_4_)_2_HPO_4_), calcium carbonate (CaCO_3_), phosphoric acid (H_3_PO_4_, 85%) and cerium ammonium nitrate ((NH_4_)_2_Ce(NO_3_)_6_) were obtained from Merck/Sigma-Aldrich (Darmstadt, Germany) at analytical grade. DMSO was used for MAPLE target preparation, and all media components and solvents for cell culture (*α*-MEM, foetal bovine serum, penicillin–streptomycin, PBS, and trypsin–EDTA) were supplied by Thermo Fisher Scientific, (Waltham, MA, USA), and handled according to the manufacturers’ instructions.

### 2.2. Synthesis of Calcium Phosphates

Brushite- (CaHPO_4_·2H_2_O), monetite- (CaHPO_4_) and cerium-doped phosphate powders used in this study were synthesised and fully characterised in our previous works [11,23], where their phase purity, crystallinity and morphology were confirmed by XRD, FTIR and SEM/EDS. Briefly, monetite and brushite were obtained by aqueous coprecipitation routes optimised to yield phase-pure dicalcium phosphates with controlled particle size, while cerium-doped phosphate was prepared by co-precipitation in basic medium followed by calcination at 500 °C or 800 °C to give the CP-Ce-500 and CP-Ce-800 powders [11], respectively. In the present work, these previously validated CaP phases were used as targets for MAPLE deposition onto electrospun nylon scaffolds without further modification.

### 2.3. Nylon Nanofibre Synthesis via Electrospinning

A nylon solution (25 wt%) was prepared by dissolving nylon 66 in formic acid, which required extended magnetic stirring and mild warming to reach complete homogeneity after 48 h. Electrospinning was performed using a commercial apparatus (Tong Li Tech TL-PRO-BM, Tong Li Nano Technology Co., Ltd., Wuhan, China), with the following standard configuration: a high-voltage power supply (0–30 kV), a syringe pump delivering polymer solution at controlled flow rates, and a grounded metallic collector [38].

The 25% nylon solution was electrospun using a 20-gauge needle with an applied voltage of 30 kV, a needle–collector working distance of 20 cm and a feed rate of 0.1 mL/h; under these conditions, uniform, bead-free nanofibres were obtained and were therefore selected as the base formulation for subsequent CaP MAPLE coatings.

### 2.4. MAPLE Deposition of Calcium Phosphate Coatings

**Figure 1:**
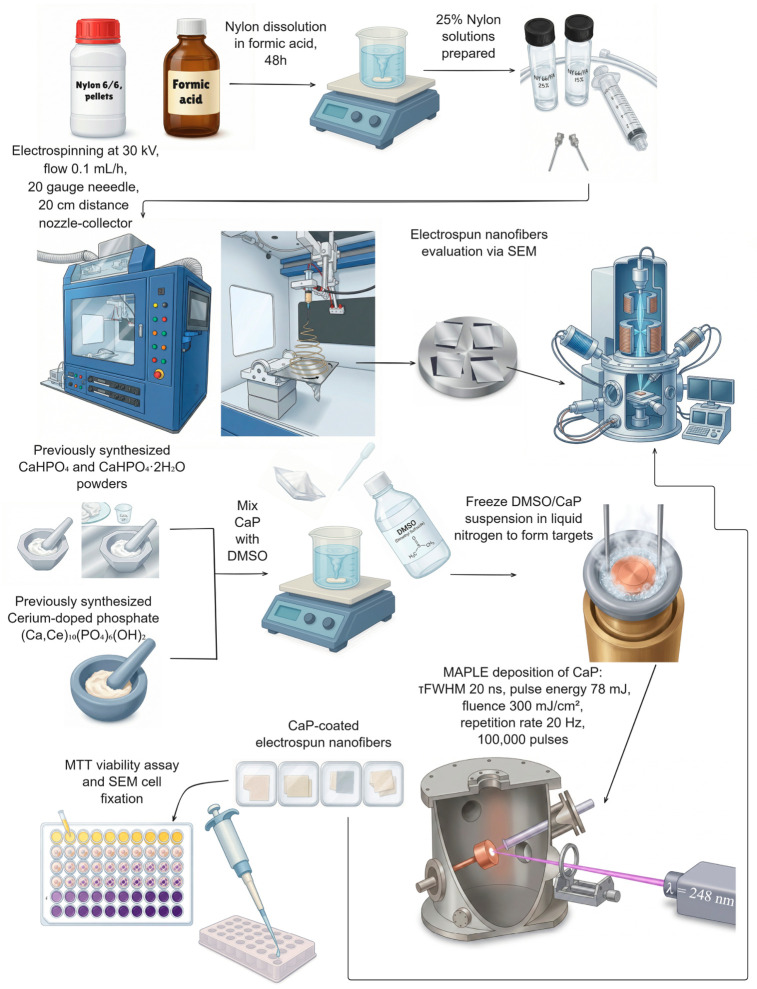
Graphical workflow of scaffold fabrication and testing (made using Illustrae [45]).

MAPLE targets were prepared by freezing the CaP suspensions in dedicated copper holders using liquid nitrogen (77 K), as shown in Figure 1. For each composition, 0.06 g of ultradispersed CaP powder was suspended in 6 mL DMSO, corresponding to a concentration of 10 g/L, and rapidly frozen to obtain a solid target.

Four distinct targets were prepared in this way: monetite-, brushite-, and cerium-doped phosphate, calcined at 500 °C and 800 °C [11]. Deposition was carried out in a stainless steel reaction chamber using a KrF excimer laser (*λ* = 248 nm, τ_FWHM_ ≈ 20 ns), operated at a fluence of 300 mJ/cm^2^ and a repetition rate of 20 Hz. The laser beam was focused on the rotating frozen target, and 100,000 pulses were applied for each deposition, with all the parameters kept constant to allow direct comparison between CaP phases. To ensure a uniform energy distribution across the laser spot, a laser beam homogeniser was used. During the MAPLE experiments, the target (maintained frozen by the cooling system) and the electrospun nylon substrates were placed 5 cm apart, in a parallel configuration, inside a high-vacuum chamber evacuated to 5 × 10^−5^ mbar. Under these conditions, the ejected CaP species condensed uniformly onto the fibres, forming thin, conformal coatings. The obtained samples are described in Table 1.

### 2.5. Characterisation Methods

#### 2.5.1. Scanning Electron Microscopy and Energy-Dispersive X-Ray Spectroscopy

The morphology of the electrospun and MAPLE-coated scaffolds, as well as of the CaP powders, was examined using a FEI Quanta Inspect F50 scanning electron microscope (Thermo Fisher Scientific, Waltham, MA, USA). The samples were mounted on aluminium stubs with carbon adhesive and sputter-coated with a 5–10 nm gold layer to reduce charging under the electron beam. Images were acquired at accelerating voltages of 10–20 kV over a wide range of magnifications to evaluate fibre continuity, coating morphology and pore architecture.

Elemental composition was assessed by energy-dispersive X-ray spectroscopy (EDS) using the detector integrated into the same equipment. Semi-quantitative analysis of peak intensities was used to compare relative CaP loading between coatings.

#### 2.5.2. Fibre Diameter Measurements

Fibre diameters were determined from representative scanning electron microscopy (SEM) images using the Fiji (ImageJ) software v. 2.17.0 [39]. For each scaffold composition, at least 50 individual fibres were randomly selected and measured. The resulting values were used to calculate mean diameter, standard deviation and diameter histograms to describe size distribution.

#### 2.5.3. Fibre Orientation Analysis

Fibre orientation was quantified on low-magnification SEM micrographs using the OrientationJ plugin [40] implemented in Fiji. Images were converted to 8-bit grayscale and minimally filtered to enhance contrast without altering fibre geometry. OrientationJ’s structure tensor analysis [41] was applied to compute the local orientation of the fibres over the whole image and to generate orientation histograms expressed as frequency versus angle (−90° to 90°). Fibre orientation was analysed to verify whether the MAPLE coating process preserved the anisotropy imposed during electrospinning. Maintaining a defined orientation is relevant for both the anisotropic mechanical response of the scaffold and for providing potential contact guidance cues to osteoblast-like cells, which can benefit from alignment similar to that found in native bone tissue. Experimental data was subsequently smoothed using a Savitzky–Golay filter (second-order polynomial) to reduce high-frequency noise while preserving peak shape. Curves were plotted in Origin 8.5.

#### 2.5.4. Fourier-Transform Infrared Spectroscopy

Chemical structure of the scaffolds was analysed by Fourier-transform infrared (FTIR) spectroscopy using a Thermo Scientific Nicolet iS50 spectrometer equipped with an attenuated total reflectance (ATR) module. Spectra were collected at room temperature in the 4000–400 cm^−1^ range, averaging 32 scans at 4 cm^−1^ resolution. The data were used to identify nylon amide bands and phosphate- and hydroxyl-related vibrations specific to monetite-, brushite- and cerium-doped phosphate.

#### 2.5.5. MC3T3-E1 Cell Culture

MC3T3-E1 preosteoblasts (American Type Culture Collection^®^, CRL-2593TM) [42,43] were cultured in Alpha Minimum Essential Medium (*α*-MEM, Gibco, Thermo Fisher, USA) supplemented with 10% foetal bovine serum, 1% L-glutamine and 1% penicillin–streptomycin under standard conditions (37 °C, 5% CO_2_, 90% humidity). The scaffolds were thermally sterilised at 120 °C for 1 h before cell seeding. Cells were seeded at a density of 40,000 cells/100 µL/sample in 12-well plates and incubated for 1 h to allow initial adhesion, after which 1 mL of fresh culture medium was added to each well. Constructs were then incubated for 2 days under standard conditions, after which cell viability assays were performed and the selected samples were prepared for morphological investigations using SEM. All the cell experiments were performed with three independent samples per condition (*n* = 3).

#### 2.5.6. MTT Viability Assay

Cell viability was assessed after 2 days (48 h) of direct contact using the MTT tetrazolium salt assay [44]. Following the incubation period, the culture medium was removed and replaced with an MTT solution prepared by diluting a 5 mg/mL MTT stock in PBS to 10% (*v*/*v*) in complete *α*-MEM; the samples were then incubated for 2 h under standard culture conditions. In this assay, metabolically active cells reduce MTT to insoluble formazan crystals, and the amount of formazan formed is proportional to cell viability [44]. After incubation, the MTT-containing medium was discarded and the formazan crystals were solubilised in DMSO.

The resulting solutions were analysed spectrophotometrically at 570 nm, and cell viability for each scaffold was calculated relative to the negative control, consisting of cells cultured on tissue-culture plastic in complete medium, which was set to 100%.

#### 2.5.7. Cell Fixation for SEM

For SEM, the culture medium was removed, and the samples were gently rinsed with PBS and fixed in 2.5% glutaraldehyde for 1 h at room temperature. After fixation, the specimens were washed with PBS and dehydrated in graded ethanol solutions (70–100%, 30 min at each step), followed by incubation in increasing HDMS:ethanol mixtures (50:50, 75:25) and finally 100% HMDS. The dried samples were subsequently mounted and sputter-coated prior to electron microscopy imaging. The cell-covered area was quantified from 1280 × 960 pixel SEM micrographs using Fiji (ImageJ). Images were thresholded (Yen method) to segment the cells from the underlying scaffold and then converted to binary. The percentage of cell-covered area was calculated as the ratio of the number of cell pixels to the total number of pixels in the image, averaged over at least three fields of view per sample.

#### 2.5.8. Statistical Analysis

The quantitative data were obtained from triplicate samples, and the results were expressed as mean ± standard deviation (SD) or mean ± standard error of the mean, as indicated in the figure captions. Statistical analysis was performed using OriginPro 8.5 (OriginLab, Northampton, MA, USA). Differences between groups were evaluated using two-tailed Student’s *t*-tests, with * *p* < 0.05, ** *p* < 0.01 and *** *p* < 0.001 considered significant.

## 3. Results

### 3.1. Nylon Nanofibre and MAPLE Deposition Characterisation

#### 3.1.1. Scanning Electron Microscopy and X-Ray Energy Dispersive Spectroscopy

**Figure 2:**
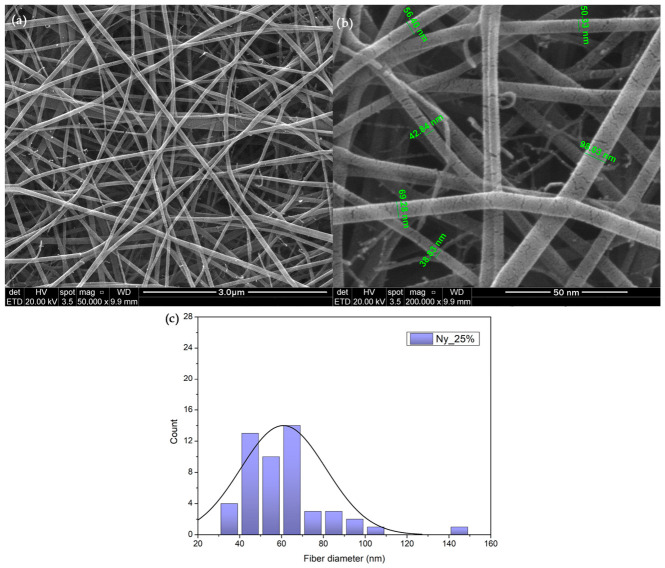
Morphological characterisation of the uncoated nylon scaffold (Ny_25%): (**a**,**b**) Low- and high-magnification SEM micrographs showing a uniform, bead-free nanofibrous network. (**c**) Fibre diameter distribution histogram for Ny_25%, with a fitted Gaussian curve illustrating the diameter range.

Electrospinning of 25% nylon solutions yielded uniform, non-woven fibre mats. The SEM analysis of the uncoated nylon scaffold (Ny_25%) in Figure 2 revealed a highly entangled mat of smooth nanofibres with a mean diameter of 62.1±23.8 nm (range 31–147 nm, *n* = 50), with only occasional thicker strands and no evidence of beading, confirming stable electrospinning conditions and preservation of high porosity. At higher magnification, individual fibres displayed well-defined contours within this nanometric range, indicative of a continuous network suitable as a base substrate for subsequent mineral functionalisation. The corresponding EDS analysis detected only the elements associated with the polymer (C, N and O from nylon), with no detectable Ca or P (as shown in Table 2), confirming that Ny_25% is a purely organic reference scaffold without intrinsic mineral content.

**Figure 3:**
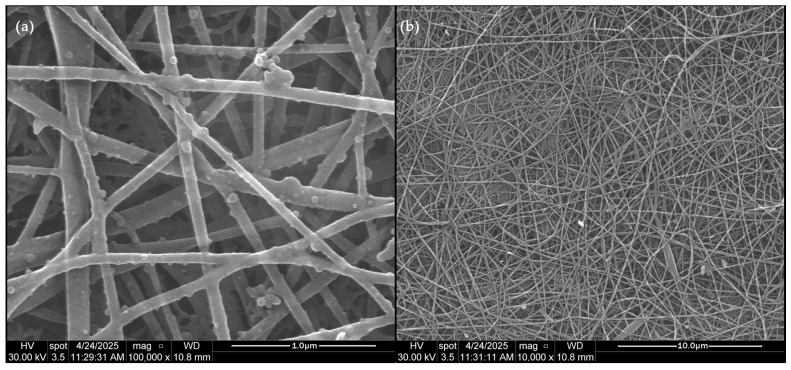
Representative SEM images of the Ny_monetite scaffold. (**a**) High-magnification view showing individual surface features (scale bar = 1 µm). (**b**) Low-magnification view illustrating the overall random fibrous network morphology (scale bar = 10 µm).

In the case of the Ny_monetite sample, the nanofibrous architecture remained clearly visible, but the SEM micrographs (Figure 3) revealed that the nylon fibres were covered by a granular coating consisting of sub-micrometric particles tightly attached to the surface. These particles formed a fine, almost conformal layer along the fibre length without generating large bridges between adjacent fibres, so that the open pore structure of the electrospun mat was largely preserved while surface roughness was markedly increased. The EDS data (Table 3) showed Ca and P besides the characteristic C, N, O signals, demonstrating successful deposition of a CaP phase over the whole analysed area and confirming that MAPLE deposition efficiently mineralised the nylon scaffold with monetite.

**Figure 4:**
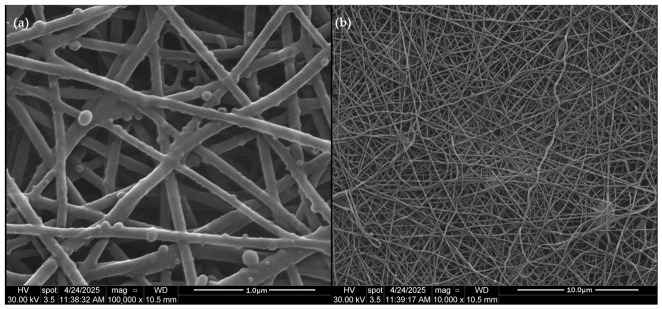
Representative SEM images of Ny_brushite. (**a**) High-magnification view showing individual surface features (scale bar = 1 µm). (**b**) Low-magnification view illustrating the overall fibrous network morphology (scale bar = 10 µm).

The SEM micrographs of Ny_brushite (Figure 4) showed a distinct coating morphology compared with monetite, with the nylon fibres partially covered by thicker, plate-like mineral formations that overlapped and occasionally formed small clusters along the filaments. While the overall fibrous structure and inter-fibre spacing were retained, local regions appeared more heavily mineralised, suggesting that brushite tends to form sheet-like deposits that enhance surface roughness but may slightly narrow pore openings compared with the finer monetite particles. The EDS analysis revealed slightly higher amounts of Ca and P in addition to the polymer- and substrate-derived elements, confirming uniform CaP deposition; the similar elemental composition but different microstructure relative to Ny_monetite indicates that the observed morphological differences arise from the intrinsic crystallisation behaviour of the brushite phase rather than from differences in overall mineral loading.

**Figure 5:**
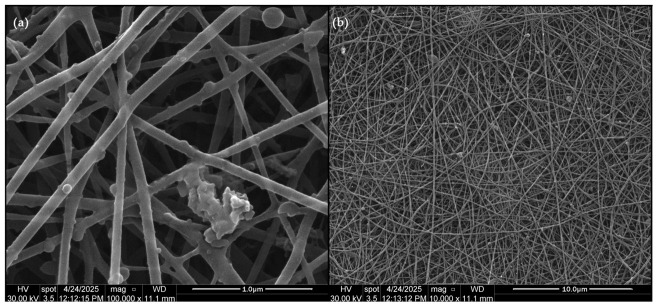
SEM images of Ny_CP_Ce_500 fibers with surface mineral aggregates. (**a**) High-magnification view showing attached particulate clusters (scale bar = 1 µm). (**b**) Low-magnification view illustrating the overall distribution of the aggregates across the fibrous network (scale bar = 10 µm).

For Ny_CP_Ce_500, the SEM imaging (Figure 5) indicated that the cerium-doped phosphate deposited from the 500 °C calcined powder formed a finely textured coating, with discrete mineral particles scattered over the nanofibre surfaces and only limited coalescence into larger aggregates. The fibres remained individually discernible, and the network kept its open, highly porous architecture, suggesting that moderate calcination temperature favours the retention of nanoscale particles that primarily modify local surface topography and chemistry without masking the underlying nylon scaffold. The EDS analysis showed Ca and P elements together with the characteristic C and O signals, confirming successful deposition of a CaP layer over the fibres while maintaining a relatively thin and dispersed coating.

**Figure 6:**
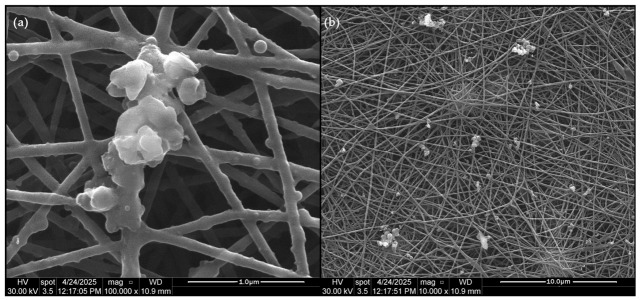
SEM images of Ny_CP_Ce_800 fibers with surface mineral aggregates. (**a**) High-magnification view showing attached particulate clusters (scale bar = 1 µm). (**b**) Low-magnification view illustrating the overall distribution of the aggregates across the ibrous network (scale bar = 10 µm).

In contrast, the Ny_CP_Ce_800 sample (Figure 6) displayed a markedly different morphology, with SEM revealing large, petal-like mineral clusters anchored to the nanofibre network and composed of densely packed particles that coalesced during calcination at 800 °C. Although individual nylon fibres could still be recognised, these compact aggregates locally spanned multiple filaments and partially obstructed the pores, indicating that higher calcination temperature promotes sintering and thickening of the cerium-doped phosphate coating, which may alter cell accessibility. The corresponding EDS revealed the highest values of Ca and P together with C, O, and N, confirming that the aggregates consist of CaP. The similar elemental composition but significantly coarser microstructure compared with Ny_CP_Ce_500 highlights the strong influence of thermal history on coating morphology and suggests a trade-off between mineral continuity and preservation of fine porosity.

#### 3.1.2. MAPLE Coated Fibre Diameter

**Figure 7:**
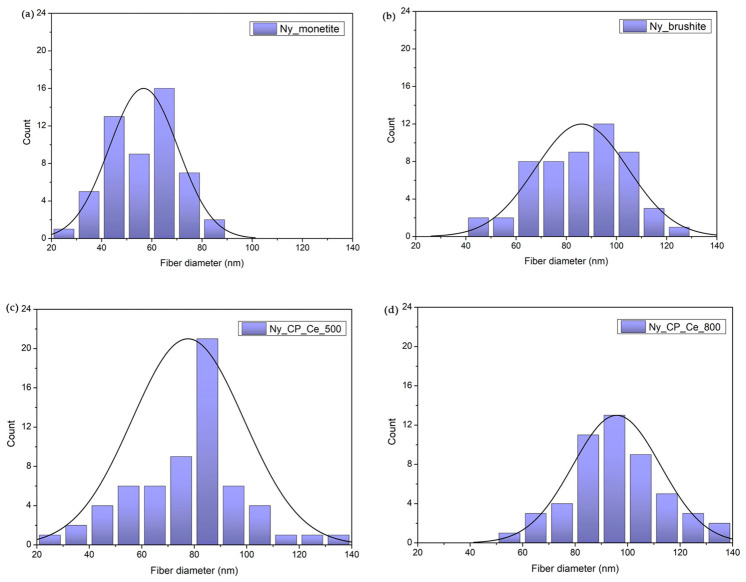
Fibre diameter distribution histograms for MAPLE-coated nylon scaffolds, with fitted Gaussian curves: (**a**) Ny_monetite, (**b**) Ny_brushite, (**c**) Ny_CP_Ce_500 and (**d**) Ny_CP_Ce_800.

The fibre diameter histograms of the MAPLE-coated scaffolds (Figure 7) show that mineral functionalisation modulates but does not radically alter these nanoscale dimensions. Ny_monetite exhibits the narrowest distribution, centred around 55–60 nm, indicating that the fine granular monetite layer preserves the original nylon diameter and even slightly reduces variability. Ny_brushite and Ny_CP_Ce_500 shift the distribution toward larger values, with most fibres in the 70–100 nm range, consistent with the formation of thicker phosphate coatings on the polymer core. Ny_CP_Ce_800 displays the largest mean diameter, approaching 100 nm and extending beyond 130 nm, which correlates with the presence of coarser aggregates along the fibres.

#### 3.1.3. Electrospun Fibre Orientation

**Figure 8:**
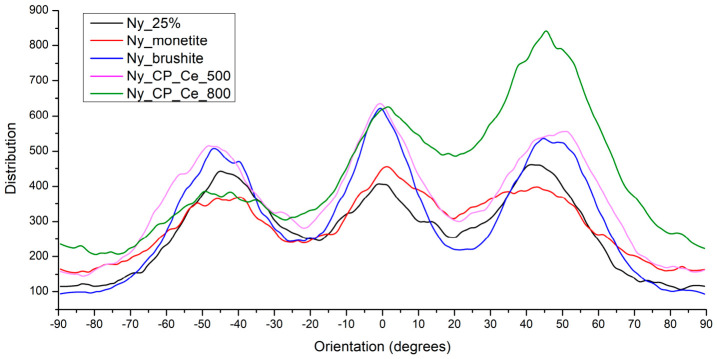
Distribution of the electrospun fibre samples based on orientation degree using the OrientationJ plugin on lower-magnification SEM micrographs.

The fibre orientation analysis (Figure 8) showed that all the samples exhibited a pronounced preferential alignment rather than a fully random distribution, with major peaks centred close to 90° (horizontal) and a secondary component near 0°, indicative of a cross-aligned nanofibre network. The alignment remained moderate, and MAPLE coating with different CaP phases did not significantly disrupt this anisotropy, as Ny_monetite, Ny_brushite, Ny_CP_Ce_500 and Ny_CP_Ce_800 all followed the same bimodal trend observed for Ny_25%. This preserved directional organisation is expected to contribute to anisotropic mechanical behaviour and may provide contact guidance cues for adherent cells along the predominant fibre direction.

#### 3.1.4. Fourier-Transform Infrared Spectroscopy

**Figure 9:**
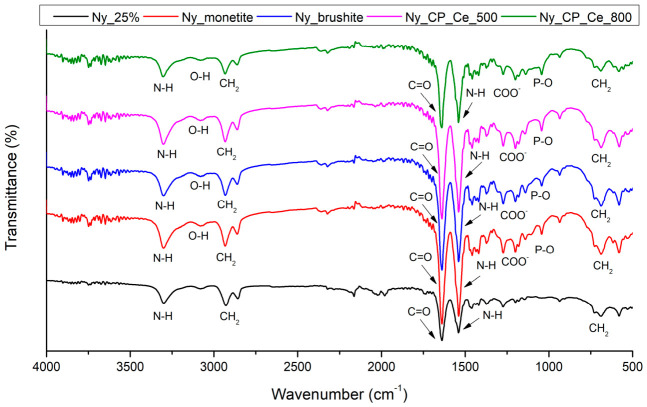
FTIR spectra of the electrospun and MAPLE-coated samples.

The FTIR analysis (Figure 9) of the electrospun nylon scaffold (Ny_25%) displayed the characteristic polyamide-6,6 bands: N–H stretching at 3300–3500 cm^−1^, C-H stretching in the range 2930–2850 cm^−1^, amide I and II at ≈1640 and ≈1540 cm^−1^, and amide III vibrations between 1260 and 1170 cm^−1^, in agreement with literature reports for nylon 6,6 [46] confirming preservation of the nylon backbone after processing. Upon MAPLE deposition of monetite (Ny_monetite), additional phosphate bands corresponding to [PO_4_]^3−^ groups emerged in the 1200–900 cm^−1^ region, together with a prominent [PO_4_]^3−^ band at ~1070 cm^−1^ and a P–OH vibration at ~880 cm^−1^, matching the reference monetite spectrum and indicating formation of anhydrous dicalcium phosphate (DCPA, CaHPO_4_). The brushite-coated fibres (Ny_brushite) exhibited a broad O–H stretching envelope between 3600 and 3100 cm^−1^ and a strong H–O–H bending mode near 1640 cm^−1^, together with characteristic [PO_4_]^3−^ vibrations, consistent with hydrated dicalcium phosphate dihydrate (DCPD, CaHPO_4_·2H_2_O), as characterised in previous work [24]. In contrast, the Ny_CP_Ce_500 and Ny_CP_Ce_800 samples showed the typical apatite [PO_4_]^3−^ bands at ≈1040–1090, 960 and 600/560 cm^−1^, confirming successful deposition of the cerium-doped phosphate without detectable degradation of the nylon substrate [11].

### 3.2. Cell Viability Evaluation

#### 3.2.1. MTT Cell Viability Assay

**Figure 10:**
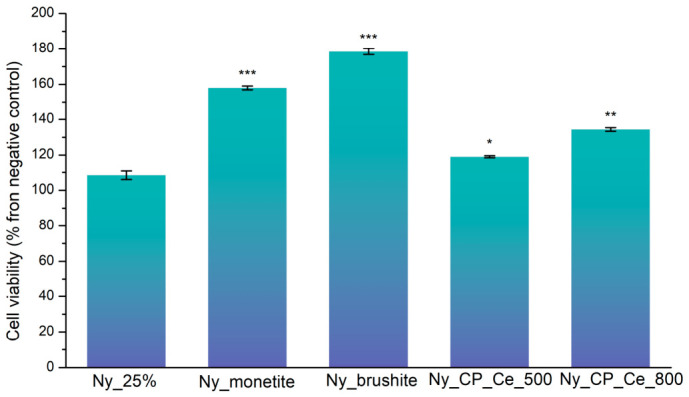
Cell viability of MC3T3-E1 preosteoblasts after 48 h culture on uncoated nylon nanofibres (Ny_25%) and MAPLE-coated scaffolds (Ny_monetite, Ny_brushite, Ny_CP_Ce_500, and Ny_CP_Ce_800), expressed as percentage relative to the tissue-culture plastic negative control (mean ± *SD*, *n* = 3; * *p* < 0.05, ** *p* < 0.01, *** *p* < 0.001 vs. negative control).

The MTT assays after 48 h (Figure 10) revealed that all the MAPLE-functionalised scaffolds maintained and enhanced MC3T3-E1 metabolic activity compared to the negative control, confirming good cytocompatibility of the coatings. The uncoated nylon (Ny_25%) showed a response similar to the negative control, with a cellular viability of about 108.4 ± 2.4% (not significant compared to the negative control). The brushite-coated fibres showed the highest viability, reaching 178.5 ± 1.7% of the negative control (*p* < 0.001), which indicates a pronounced stimulatory effect on preosteoblast metabolism. Monetite- and cerium-doped phosphate coatings also increased cell viability above the negative control (*p* < 0.05), but to a lesser extent, suggesting that CaP chemistry and microstructure modulate the early osteogenic response. The lower, but still statistically significant, cellular metabolic values measured for Ny_CP_Ce_500 (119 ± 0.6%, *p* = 0.04) and Ny_CP_Ce_800 (134.3 ± 1%, *p* = 0.003) in comparison to the negative control are consistent with their finer or more aggregated morphologies and indicate that the contribution of cerium does not translate into an acute boost of osteoblastic metabolism at 48 h.

#### 3.2.2. Cell Distribution via SEM Imaging

**Figure 11:**
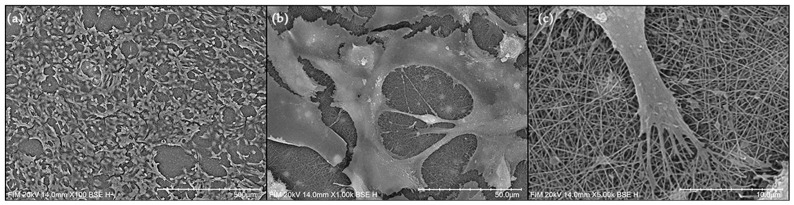
SEM micrographs of MC3T3-E1 preosteoblasts cultured for 48 h on the Ny_25% scaffold. (**a**) Low-magnification view showing overall cell coverage across the nanofibrous mat (scale bar = 500 μm). (**b**) Intermediate-magnification image illustrating cell spreading and morphology on the scaffold surface (scale bar = 50 μm). (**c**) High-magnification image highlighting close interactions between cellular processes and individual nanofibres (scale bar = 10 μm).

For the uncoated nylon scaffold (Ny_25%), SEM imaging of MC3T3-E1 (Figure 11a) preosteoblasts after 48 h revealed a continuous but relatively sparse cellular layer, with cells displaying mainly flattened morphologies and limited spreading (Figure 11b,c) compared to the mineral-coated samples. Quantitative image analysis showed a cell-covered area of 41.50%, consistent with the presence of numerous bare regions of exposed fibres at low magnification, indicating that the pristine nylon surface anchored cells at multiple contact points to the nanofibres, with short filopodia bridging neighbouring strands and following the underlying fibre orientation.

**Figure 12:**
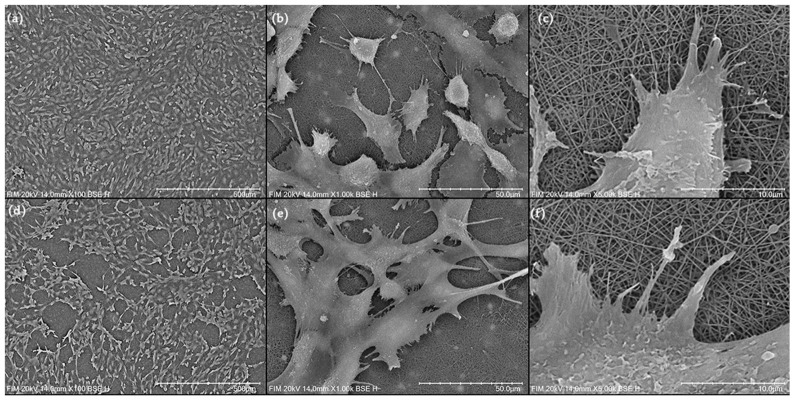
SEM micrographs of MC3T3-E1 preosteoblasts cultured for 48 h on Ny_monetite (**a**–**c**) scaffolds at increasing magnifications: (**a**) scale bar = 500 μm, (**b**) scale bar = 50 μm, and (**c**) scale bar = 10 μm. Cells on Ny_brushite (**d**–**f**) scaffolds at the corresponding magnifications, (**d**) scale bar = 500 μm, (**e**) scale bar = 50 μm, and (**f**) scale bar = 10 μm.

On the monetite-functionalised fibres (Ny_monetite), the SEM images (Figure 12a) revealed dense cellular coverage after 48 h, with preosteoblasts forming an almost continuous layer that closely followed the underlying nanofibre topography. This observation was confirmed by a high cell-covered area of 66.32%, indicating substantially improved colonisation relative to Ny_25%. At higher magnification (Figure 12b,c), the cells exhibited extensive spreading and numerous filopodial extensions bridging adjacent fibres, suggesting strong focal adhesion and intimate contact with the monetite particles, in line with the elevated MTT metabolic activity measured for this composition. Ny_brushite scaffolds also supported robust MC3T3-E1 attachment, with the SEM micrographs (Figure 12d) showing confluent cell monolayer regions interspersed with small pores corresponding to the underlying brushite-coated fibre bundles. The measured cell-covered area for Ny_brushite was 43.98%, comparable to Ny_25% and lower than Ny_monetite despite its favourable MTT result. This apparent discrepancy arises from the heterogeneous distribution of cells, which tend to form overlapping clusters and locally high cell densities in preferred regions (as shown in Figure 12e,f), leaving neighbouring areas with fewer cells and thus reducing the overall projected coverage in the analysed fields.

**Figure 13:**
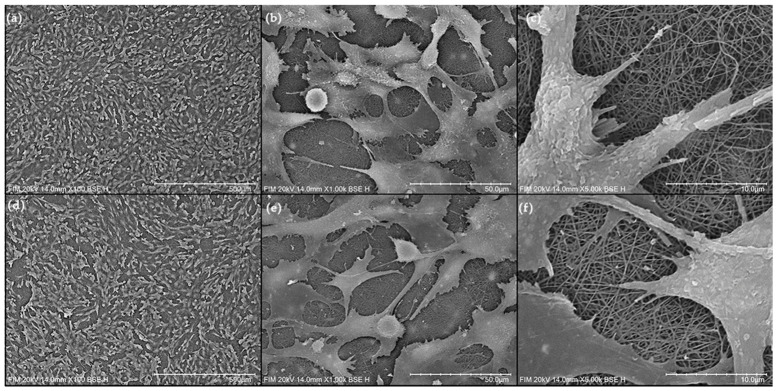
SEM micrographs of MC3T3-E1 preosteoblasts cultured for 48 h on Ny_CP_Ce_500 (**a**–**c**) scaffolds at increasing magnifications: (**a**) scale bar = 500 μm, (**b**) scale bar = 50 μm, and (**c**) scale bar = 10 μm. Cells on Ny_CP_Ce_800 (**d**–**f**) scaffolds at the corresponding magnifications, (**d**) scale bar = 500 μm, (**e**) scale bar = 50 μm, and (**f**) scale bar = 10 μm.

On Ny_CP_Ce_500, the SEM micrographs (Figure 13a) revealed a continuous but somewhat more heterogeneous cellular distribution, with regions of well-spread cells interspersed with sparsely populated areas where the cerium-doped phosphate-coated fibres remained visible. Nevertheless, image analysis indicated the highest cell-covered area among the tested coatings, reaching 72.51%, which confirms that this formulation supports extensive surface colonisation, in agreement with its good viability values. At high magnification (Figure 13b,c), adherent cells extended filopodia that anchored to individual fibres and appeared to probe the nanoscale phosphate particles, suggesting that this coating maintains good cytocompatibility. In contrast, the Ny_CP_Ce_800 samples displayed more discontinuous cell coverage, with SEM images (Figure 13d) revealing clusters of preosteoblasts separated by regions dominated by large apatitic aggregates and exposed fibres. The corresponding cell-covered area was 60.76%, lower than Ny_CP_Ce_500 but still higher than Ny_brushite. The higher-magnification images (Figure 13e,f) showed cells that were still able to attach and spread but often wrapped around or perched on top of the coarse mineral clusters, indicating that excessive aggregate size and surface roughness may hinder uniform colonisation.

## 4. Discussion

Electrospun nylon nanofibres functionalised with bioactive CaP phases via MAPLE represent a versatile platform for bone tissue engineering. Systematic variation in phosphate chemistry (monetite-, brushite-, and cerium-doped phosphate) and processing parameters revealed the brushite-coated fibres to have the most bone cell-stimulating properties, enhancing preosteoblasts’ metabolism to about 178% of the control levels. The gentle MAPLE deposition process preserved fibre diameter and three-dimensional architecture while enabling mineral deposition across the nanofibre network. The fact that MAPLE deposition did not significantly alter the bimodal orientation distributions indicates that mineral functionalisation can be achieved without disrupting the underlying fibrous anisotropy.

The distinct morphologies observed for brushite, monetite, and cerium-doped phosphate reflect differences in particle size, crystallinity, and interaction with the polymeric substrate. Monetite fine, uniform granular coating provides maximal surface area for cellular interaction while minimising aggregate bridging between adjacent fibres, preserving three-dimensional porosity. Brushite sheet-like morphology, while promoting roughness, may create regions of local fibre fusion that reduce pore accessibility. Cerium-doped phosphate phase-dependent morphology [11] illustrates how thermal processing modulates material behaviour: the moderate temperature (500 °C) preserves fine particle size and dispersed distribution, while aggressive calcination (800 °C) promotes sintering and coalescence, potentially limiting cellular accessibility to mineral surfaces.

The reduced measured performances of the cerium-doped phases at 48 h suggest that additional functionalities induced by Ce ions do not necessarily improve the bone cells’ response as compared with monetite. Given the thinness of the deposited layer and the low overall cerium content, these coatings are not expected to induce cytotoxic effects based on concentration alone, but extended culture periods, antimicrobial evaluation and in vivo implantation studies will be necessary to fully evaluate cerium-doped phosphate utility [47].

The preservation of fibre diameter and structural integrity following MAPLE deposition validates this approach as a gentle functionalisation method compatible with nanofibrous substrates. Unlike conventional pulsed laser deposition [48] or thermal spraying techniques that can alter or destroy the underlying fibre architecture [29], the MAPLE deposition process and room-temperature substrate exposure maintain scaffold porosity and mechanical continuity [35]. Similar behaviour has been reported for MAPLE coatings on flexible polymers and three-dimensional architectures, where mineral, polymeric or hybrid films [28] are deposited without compromising substrate morphology or mechanics [34]. In the presented system, this compatibility enables a modular design in which electrospun nylon mats are first optimised solely for structural performance [18] and subsequently functionalised with diverse bioactive CaP [36] or composite phases [14], thereby reducing process complexity and expanding the accessible material design space.

The developed phosphate-coated nylon fibre scaffolds address several critical design criteria for bone regeneration [49]: micro-architecture mimicking native ECM porosity, bioactivity through CaP deposition, mechanical resilience from nylon’s inherent strength, and customisability through modular MAPLE deposition. The hierarchical fibre organisation combined with nanoscale mineral coatings creates a multi-scale architecture conducive to osteogenic differentiation [3] and mineralisation. These findings align with recent efforts to design hierarchical or gradient scaffolds for bone regeneration, such as gel-based systems with spatially controlled mineral content [50] and hierarchical porous polymer/amorphous calcium phosphate membranes [51]. Importantly, although calcium phosphate ceramics are intrinsically brittle, confining them to thin, conformal layers on a ductile fibrous substrate mitigates this limitation, allowing the scaffold to retain overall flexibility while still presenting a bioactive mineral interface to cells.

Future investigations should include: extended cell culture assays (14, 21 days) to evaluate proliferation and osteogenic marker expression (alkaline phosphatase and osteocalcin) [52]; incorporation of osteoinductive factors (bone morphogenetic proteins and growth factors) via dual MAPLE deposition or subsequent chemical functionalisation; optimisation of coating thickness and porosity through laser parameter variation; mechanical evaluations and manufacturing scale-up and regulatory pathway assessment for clinical translation.

These findings establish a rational design framework for customisable, bioactive fibrous scaffolds poised for clinical translation in bone regeneration and other hard tissue engineering applications.

## 5. Conclusions

This study demonstrates that combining electrospinning with matrix-assisted pulsed laser evaporation enables precise, phase-specific mineral functionalisation of nylon nanofibres without compromising their nanoscale architecture. Monetite-, brushite- and cerium-doped phosphate could all be deposited as conformal coatings, yet their morphology and biological performance diverged markedly, underscoring the importance of calcium phosphate chemistry in scaffold design. The brushite-coated fibres were particularly effective, generating a homogeneous granular layer that preserved high porosity and produced the highest early preosteoblast metabolism, highlighting this phase as a strong candidate for promoting rapid osteogenic activation at the implant interface. The monetite coatings enhanced surface roughness and supported robust cell adhesion but showed comparatively lower metabolic stimulation, suggesting that their lower solubility and tendency to form thinner structures may moderate short-term bone cell signalling. The cerium-doped phosphate provided a route to introduce rare-earth functionality; however, the strong sintering and aggregate growth observed at 800 °C indicate that careful control of calcination is required to balance the well-known antibacterial or antioxidant benefits against potential reductions in accessible surface area.

From a processing standpoint, the results confirm that MAPLE can uniformly deposit inorganic phases into nanofibrous scaffolds while maintaining fibre diameter, orientation and mechanical continuity, which are often compromised by conventional high-energy coating methods. This compatibility opens the possibility of decoupling scaffold fabrication from surface functionalisation [53]: electrospinning can be optimised solely for structural and mechanical performance, after which MAPLE can independently tune the mineral phase, loading and distribution. Such modularity is particularly attractive for clinical translation, where different calcium phosphate chemistries, dopants or drug payloads may be required for distinct indications or patient populations [54].

The phosphate-coated nylon scaffolds developed here meet several central criteria for next-generation bone graft substitutes: they mimic the fibrous architecture of the native extracellular matrix, incorporate bioactive mineral phases capable of delivering osteoconductive and potentially osteoinductive cues, and rely on a processable synthetic backbone. Together, these attributes create a multi-scale environment that can support cell attachment, guide tissue organisation and accommodate physiological loading. Looking ahead, extending in vitro assays to longer time points and relevant osteogenic markers, followed by *in vivo* evaluation in critical-size defect models, will be essential to confirm whether the early advantages observed for monetite translate into superior bone formation and integration. Parallel optimisation of MAPLE, coating thickness and dopant content could ultimately position this platform as a flexible manufacturing route for mineralised fibrous scaffolds aimed at repairing challenging defects in craniofacial and dental applications.

## Figures and Tables

**Table 1:** Sample abbreviations and descriptions.

Sample	Description
Ny_25%	Electrospun nylon scaffold (25 wt% solution), uncoated
Ny_monetite	Ny_25% scaffold coated by MAPLE with monetite (CaHPO_4_)
Ny_brushite	Ny_25% scaffold coated by MAPLE with brushite (CaHPO_4_·2H_2_O)
Ny_CP_Ce_500	Ny_25% scaffold coated by MAPLE with cerium-doped phosphate calcined at 500 °C
Ny_CP_Ce_800	Ny_25% scaffold coated by MAPLE with cerium-doped phosphate calcined at 800 °C

**Table 2:** Semi-quantitative EDS analysis of the reference sample.

Sample	C (%)	N (%)	O (%)
Ny_25%	78.2	9.2	12.6

**Table 3:** Semi-quantitative EDS analysis of the MAPLE-coated samples.

Sample	C (%)	N (%)	O (%)	P (%)	Ca (%)
Ny_monetite	75.9	7.1	9.2	4.3	3.5
Ny_brushite	68.4	3.2	5.6	5.6	4.1
Ny_CP_Ce_500	47.3	6.6	12.2	12.2	8.0
Ny_CP_Ce_800	42.0	4.8	15.3	15.3	17.0

## Data Availability

Data is contained within the article.

## Abbreviations

The following abbreviations are used in this manuscript:

MAPLE	Matrix-Assisted Pulsed Laser Evaporation
CaP	Calcium Phosphate
SEM	Scanning Electron Microscopy
EDS	Energy-dispersive X-ray Spectroscopy
FTIR	Fourier-Transform Infrared Spectroscopy
PBS	Phosphate-buffered Saline
MTT	(3-(4,5-Dimethylthiazol-2-yl)-2,5-Diphenyltetrazolium Bromide)
HDMS	Hexamethyldisilazane
DMSO	Dimethyl Sulfoxide
